# Material Shifts and Trends: A Comprehensive Dataset of European Car Fleet Composition, 1980–2050

**DOI:** 10.1038/s41597-026-07898-6

**Published:** 2026-07-17

**Authors:** Matthias Rösslein, Manuele Capelli, Max Tippner, Franziska Maisel, Kirsten Remmen

**Affiliations:** 1 Laboratory for Nanomaterials in Health, Swiss Federal Laboratories for Materials Research and Testing (Empa), St. Gallen, Switzerland; 2 Technology and Society Laboratory, Swiss Federal Laboratories for Materials Research and Testing (Empa), St. Gallen, Switzerland; 3 Fraunhofer-Institute for Reliability and Microintegration, Dept. Environmental and Reliability Engineering, Berlin, Germany

## Abstract

The transition to electrified drivetrains fundamentally reshapes material demand and future secondary raw material supply. However, existing vehicle composition data are often fragmented or proprietary. This study presents a harmonized, open-access dataset detailing the material and elemental composition of the European passenger car fleet (M1 category) from 1980 to 2050. Using integrated top-down and bottom-up methodologies, we provide high-resolution data for six drivetrain types and 13 vehicle segments, including crossover utility vehicles and battery-electric models. The dataset links products, components, materials, and elements (p-c-m-e), with standardized alloy specifications and quantified uncertainty. Technical validation confirms high internal consistency, with Pearson correlation coefficients (r) between independent modeling approaches exceeding 0.96 for total metal, aluminum, and iron/steel content. This dataset supports robust stock-and-flow modeling and recoverability analysis beyond mass-based indicators. By aligning with emerging regulatory tools like Digital Product Passports, it enables evidence-based resource strategies and circular economy planning for critical and strategic raw materials.

## Background & Summary

The effective management of raw material supply and demand requires comprehensive, reliable data on secondary raw material stocks and flows throughout their life cycles^1,2^. A critical aspect of the assessment of whether materials are recoverable is not only identifying which elements are present in products but also understanding their material composition and the components in which they are embedded^3–5^. This level of detail is essential for evaluating recoverability. However, existing data on material composition are often fragmented, incomplete, and not structured in a way that enables systematic recoverability assessments^1,2,4^.

One significant source of secondary raw materials is end-of-life vehicles (ELVs)^6–8^. Strategic resource management depends on understanding how the material composition of vehicles has evolved over time and how it will continue to change^9,10^. This is particularly relevant considering the transition from internal combustion engine vehicles to battery-electric vehicles, which fundamentally alters material demand and the corresponding secondary raw material supply landscape^11–13^. Specifically, the demand of critical raw materials (CRMs) as defined by the European Commission is impacted by this shift^14–16^. Since the material composition of vehicles placed on the market constitutes the essential starting point for any subsequent modelling approach^17,18^, this study concentrates on this aspect and does not address the specific requirements of modelling end-of-life vehicle processes.

Limited data accessibility remains a critical barrier to advancing research in this field. Information on material composition and flows is frequently proprietary or inaccessible, which restricts transparency and constrains comprehensive system-level analysis. Available datasets are dispersed across heterogeneous sources and lack standardization, resulting in inconsistencies and poor comparability^1,2,18^. This fragmentation complicates robust stock and flow modelling, undermines the reliability of projections, and ultimately limits the development of evidence-based resource recovery strategies^1,7^.

**Fig. 1:**
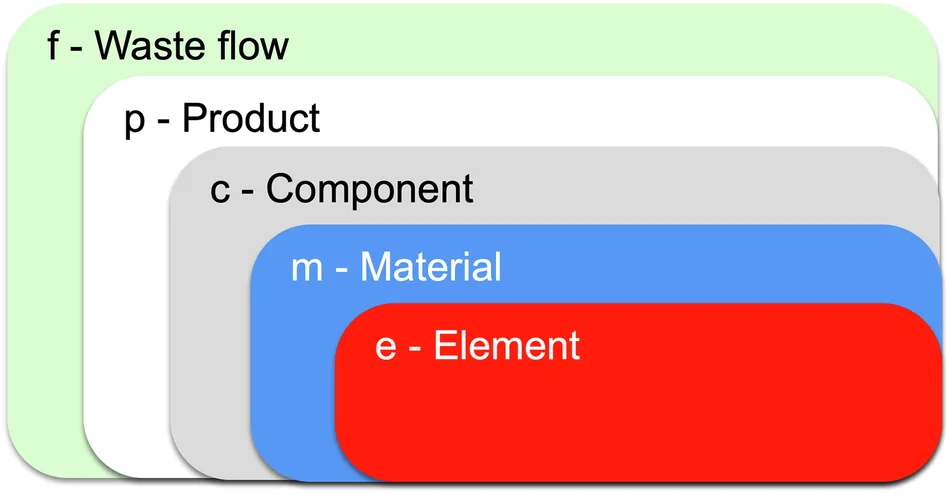
Layered approach for describing material composition, linking waste flows (**f**) to products (**p**), components (**c**), materials (**m**), and elemental content (**e**).

To address these challenges, we present a dataset that provides structured, harmonized, and openly accessible information on the composition of vehicles, including materials, alloys, and elements^2,4^. Through a combined top-down and bottom-up approach, the model integrates detailed composition data while also incorporating electric vehicles (battery electric vehicles, plug-in hybrid vehicles, and hybrid vehicles) into the dataset^17,19^. In the top-down approach, the overall material composition of an average vehicle is considered in aggregate form, providing a broad picture of the stocks and flows at system level. In contrast, the bottom-up approach breaks the vehicle down into its individual components and materials and then aggregates these back together to represent the whole vehicle. By combining these complementary methods, the model benefits from both the holistic perspective of top-down data and the granularity of bottom-up data, resulting in a more consistent and robust representation of vehicle composition^17^. This approach ensures that each element of interest is linked to a specific material classification within the dataset, enabling a more granular assessment of recoverability beyond mass-based indicators (see Fig. 1)^4,20^.

The objective is to create a standardized composition dataset for vehicles, covering components, materials, and elements in a structured format. By providing detailed insights into CRM content and associated material properties, this dataset has the potential to support policy interventions, particularly in developing material and element-specific indicators^6,15,20^.

Additionally, the model is designed for seamless integration with evolving regulatory frameworks, such as the Digital Product Passport (e.g. Battery passport)^13,18,21,22^ and other product related reporting systems. This adaptability ensures that as new data emerge, they can be incorporated efficiently, making the dataset as well as the underlying model a valuable tool for long-term resource planning and circular economy strategies^21–25^.

## Methods

### Scope

The scope of this study is defined by the European Union’s M1 category regulation, encompassing passenger vehicles with up to eight seats in addition to the driver, regardless of drivetrain technology^21^. Within the framework of the FutuRaM project, the primary objective is to characterize the material and elemental composition of the European fleet to enhance the recovery of secondary and critical raw materials (CRMs). By establishing a high-resolution baseline of vehicle material composition, this research supports the development of advanced recycling strategies and circular economy pathways for the automotive sector.

The material analysis focuses predominantly on the evolution of aluminum alloys and their potential substitution by magnesium alloys in premium segments, alongside a detailed taxonomy of iron and steel grades^1,9,10^. While the study incorporates the mass contributions of electronic control units (ECUs)^26^ and battery packs, these complex assemblies are treated as aggregated components. This level of granularity aligns with the FutuRaM project’s focus on current industrial recycling practices, where high-value or hazardous components are typically removed during a pre-shredder disassembly phase before the remaining vehicle structure undergoes mechanical fragmentation^1,27,28^.

To capture the technological diversity of the market, vehicles are classified according to two principal dimensions: drivetrain technology (ICEV, BEV, HEV, PHEV, and FCEV)^29,30^ and market segmentation (A–F and J-segment crossovers)^31,32^. Data spanning 1980 to 2025 were compiled and interpolated to produce annual composition values, with projections extending to 2030 based on observed industry trajectories^10,33–37^. For the 2030–2050 period, material intensities are held constant to distinguish empirical trends from long-term assumptions. This structured approach ensures that the resulting dataset is optimized for analyzing the material flows and recycling potential of the modern and future European automotive fleet.

### Data classifications

Prior to detailing the data analysis and completion process, we define and describe the four vehicle layers - Product, Component, Material, and Element (Fig. 1) - which form the basis of the data classification scheme.

#### Product: Drivetrain technology

Vehicles are classified into six different drivetrain technologies (see Table 1): Petrol, diesel, which both belong to the category of ICEV, Battery Electric Vehicles (BEV), Plug-in Hybrid Electric Vehicles (PHEV), and Hybrid Electric Vehicles (HEV). Other technologies with very low EU market shares in 2025 (e.g., LNG, fuel cells) are grouped as “Others” and not handle separately^30,34,38–41^.

**Table 1:** Classification and description of major drivetrain technologies.

Drivetrain technology	Description
Petrol	Belongs to Internal Combustion Engine Vehicle (ICEV)^106^ and it is powered by petrol engines.
Diesel	Belongs to ICEV and it is powered by diesel engines.
**BEV**	Battery Electric Vehicle: It is a purely electric system powered exclusively by motor(s) that draw their energy from a rechargeable high-voltage battery.
**PHEV**	Plug-in Hybrid Electric Vehicle: It is a hybrid system that combines an internal combustion engine with an electric motor and a large battery that can be charged both while driving and via an external power source.
**HEV**	Hybrid Electric Vehicle: The drive system is a combined drive system that combines an internal combustion engine with at least one electric motor and a battery that is charged exclusively by the engine or by regenerative braking.
**Others**	Includes all other existing but niche drivetrain technologies, such as LPG or hydrogen fuel cell vehicles.

#### Product: Vehicle classification

Passenger vehicles in Europe are classified into segments A–F based on vehicle size and characteristics (see Table 2). The segmentation framework was progressively harmonized across national markets and integrated into European regulatory and statistical practice^29–32,39,42–46^. It is now widely applied in EU policy documents, market analyses, and regulatory contexts as a standardized reference for categorizing passenger vehicles.

**Table 2:** Classification of passenger car segments with descriptions and representative models.

Segment	Description	Examples
**A**	Mini city cars with compact dimensions for urban use.	Fiat 500, VW Up!, Toyota Aygo X, Kia Picanto, Hyundai i10
**B**	Small cars (superminis) with increased space and comfort.	Ford Fiesta, VW Polo, Renault Clio, Peugeot 208, Opel Corsa
**C**	Compact cars balancing size, efficiency, and practicality.	Toyota Corolla, VW Golf, Honda Civic, Hyundai i30, Mazda 3
**D**	Mid-size cars with greater space and performance, suited for families.	VW Passat, Toyota Camry, Mazda 6, Honda Accord, BMW 3 Series
**E**	Premium, large-format executive cars designed for high-end business travel.	BMW 5 Series, Mercedes-Benz E-Class, Audi A6, Volvo S90
**F**	Flagship luxury cars offering maximum scale, opulence, and peak performance.	BMW 7 Series, Mercedes-Benz S-Class, Audi A8, Lexus LS

The main classification criteria for passenger vehicles are listed below:

1. **Size and Dimensions:** Vehicle length, width, and height (thresholds adjusted over time).
2. **Purpose and Use:** Intended application (e.g., urban, family luxury).
3. **Equipment and Features:** Level of equipment, comfort and technology.
4. **Market Position:** Price segment and target market.

The proliferation of the Crossover Utility Vehicle (CUV) has fundamentally redefined automotive segmentation by blurring the traditional dichotomy between sedans and Sport Utility Vehicles (SUVs). While CUVs adopt the “two-box” morphology and elevated ride height of SUVs, they utilize a car-based unibody construction rather than a truck-based body-on-frame architecture. This synthesis allows CUVs to offer SUV-like cargo utility and passenger space while maintaining the fuel efficiency and dynamic on-road behavior of conventional passenger cars.

This form factor has become the de facto standard for Battery Electric Vehicles (BEVs), largely driven by the “skateboard” chassis^38,47–49^. By housing the battery pack flat between the axles, this modular platform eliminates internal combustion packaging constraints, enabling a flat cabin floor and maximizing the interior volume inherent to the CUV body style. Consequently, the European J-segment (crossovers/SUVs) has been subdivided into categories JA through JF (e.g., JA for small urban crossovers, JC for medium), mirroring the traditional sedan-based A, B, C, D, E and F segments.

While all these vehicles fall under the European Union’s M1 classification for passenger cars^30^, traditional statistical frameworks - such as those used by Eurostat - remain heavily reliant on weight-based categories. Because the mass of battery packs often pushes BEVs into higher weight brackets regardless of their physical size, there is an urgent need to modernize these classifications to reflect the new drivetrain technologies and the crossover-dominated market landscape.

#### Component

Vehicle architecture is analyzed through a hierarchical decomposition into component families - major structural groupings such as the body-in-white, powertrain, and chassis^50^. This study utilizes a multi-level taxonomy where Level 1 categories align with contemporary manufacturer classifications (Table 3), while a broader Level 0 categorization is maintained to ensure backward compatibility with legacy datasets^51^.

**Table 3:** Mapping of major component categories from the previous dataset (Level 0) to the new component classification based on industrial standards (Level 1).

Component Category - Level 0	Component category - Level 1
**General Components**	Body in White (BIW)
	Brakes
	Chassis
	Closures
	Driveline
	Powertrain
	Steering
	Thermal Management
	Transmission
	Trim
	Wheels
**Power Electronics (xEVs)**	Power Electronics
**Embedded Electronic Components**	Controllers
	Actuators
	Headlights
	Cables
**Catalytic Converter (ICEV)**	Catalytic Converter
**Traction Battery (xEVs)**	Traction Battery ^*)^
**Rest**	Rest ^*)^

^*)^Traction batteries and remaining materials are represented only at the component level (Level 1), due to limited granularity in material data. This ensures consistency in determining the total vehicle weight.

The Level 0 framework, inherited from previous research, presents specific structural limitations; while it includes a dedicated drivetrain category for BEVs, it lacks a corresponding separate classification for ICEVs. Despite this inconsistency, the structure was retained to allow for longitudinal material composition analysis. To resolve these inadequacies, this study introduces a novel approach that systematically decomposes all relevant component families. This methodology enables a granular analysis of specialized sub-components - such as permanent magnets and copper windings - ensuring that total vehicle mass is accurately accounted for while replacing legacy categories with a more robust, drivetrain, and electronics agnostic taxonomy. It is called bottom-up approach.

#### Material & Element composition

**Fig. 2:**
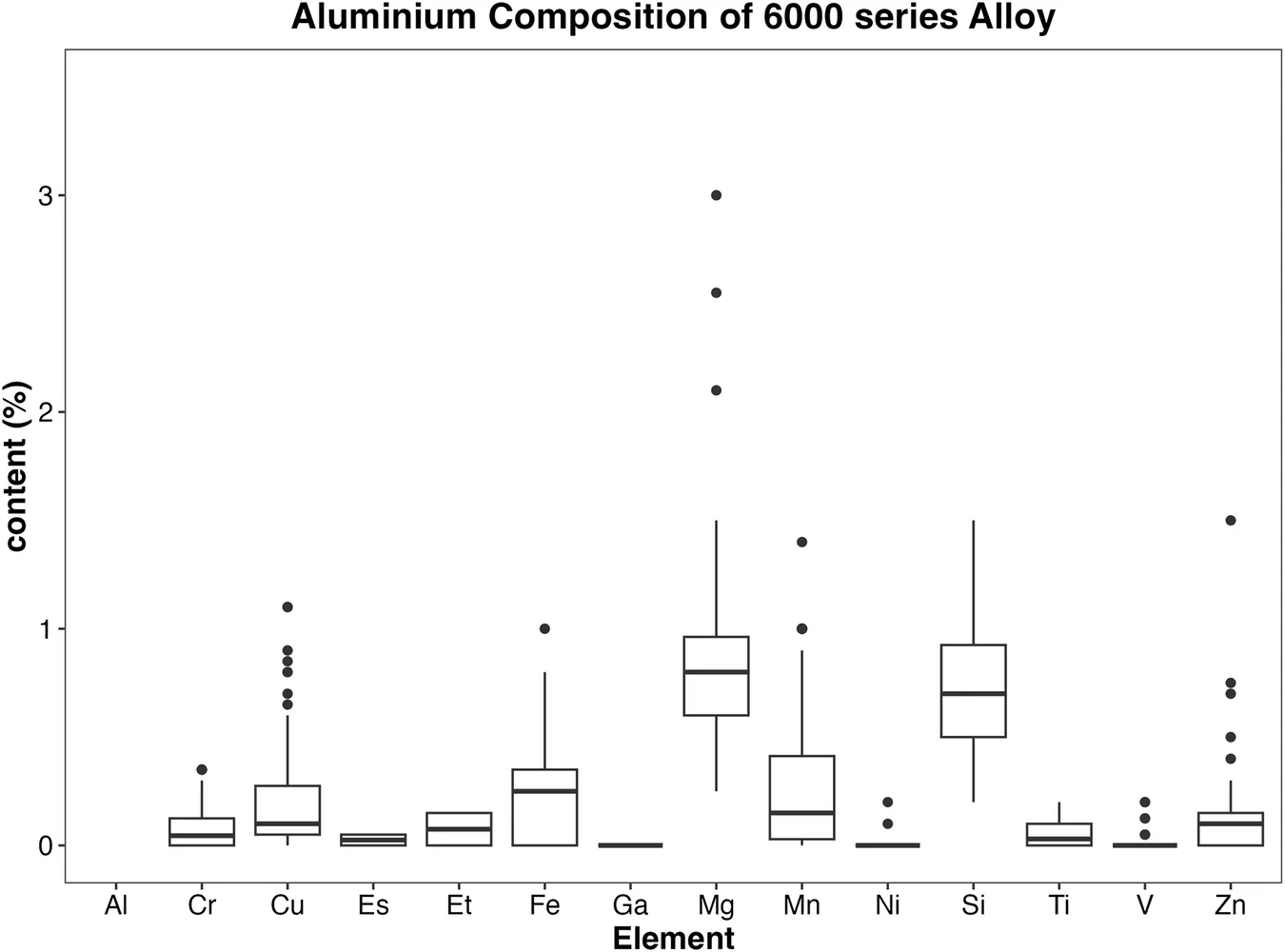
Overall elemental composition of the EN 6xxx aluminum alloy series, showing elevated concentrations of magnesium (Mg) and silicon (Si), the primary contributors to its characteristic properties. Minor alloying elements, including copper (Cu), iron (Fe), manganese (Mn), and zinc (Zn), also influence the final material behavior. It denotes the contribution of individual minor elements, while it represents their total combined contribution.

Vehicle component families are decomposed into subcomponents characterized by specific metal alloys, with elemental compositions derived from international standards (ISO, EN, ASTM, SAE). For aluminum, the Aluminum Association (AA) designation system (ANSI H35.1) provides the chemical limits for wrought (1xxx–8xxx) and cast (xxx.x) alloys^52–56^. The 6xxx series (Al-Mg-Si) is particularly central to automotive engineering due to its optimal balance of weldability, extrusion properties, and strength for crash-relevant structures (see Fig. 2).

In this study, the elemental makeup of each alloy is estimated by averaging the specified minimum and maximum concentration values, with the base metal constituting the balance. In automotive manufacturing, 5xxx, 6xxx, and 7xxx series aluminum alloys are selected based on component function and forming processes, such as rolling or forging^10^. Due to manufacturer-specific variations, representative alloys from each series were selected, and their mean elemental profiles were used to calculate material contributions. While global standards facilitate the analysis of iron and steel, proprietary data for advanced high-strength steels necessitates reliance on the most comprehensive published composition data.

Specialized component families - including electronics, traction motors (BEV/HEV/PHEV), and catalytic converters (ICEV) - are analyzed at a granular level, focusing on Critical Raw Materials (CRMs), Strategic Raw Materials (SRMs), and Platinum Group Elements (PGEs). For hybrid drivetrains, the analysis accounts for additional generator mass. Conversely, non-metallic materials and battery packs are treated as aggregate units to maintain a consistent total vehicle mass balance. These averaged elemental profiles were integrated into a comprehensive vehicle material composition dataset (p-c-m-e), resulting in a high-resolution structure containing between 148,000 and 172,000 entries per drivetrain, available in Excel and CSV formats for statistical analysis^57^.

### Data collection

Detailed information regarding passenger vehicle composition is often proprietary, as material innovations are closely guarded by Original Equipment Manufacturers (OEMs)^58^ for competitive advantage. While the (IMDS)^59^ serves as a centralized repository for industry stakeholders and regulatory compliance, its data remains inaccessible to the broader research community.

To overcome these data limitations, this study employed two complementary and mutually validating methodologies to derive representative average compositions across vehicle segments (A-F) and crossover categories (JA-JF). The top-down approach assessed composition based on total material content and the overall utilization of various alloys within a vehicle. Conversely, the bottom-up approach derives composition by analyzing the material shares within specific component families and sub-components. This hierarchical analysis is structured through a multi-level data descriptor - product, component, material, and element (p-c-m-e) - enabling the systematic tracking of elemental flows from the vehicle level down to specific alloy constituents.

A critical aspect of this methodology is the initial use of a “standard car” model, a composite architecture derived from industry reports that represents a weighted average of all vehicle segments^10,34,36,37,60^. This model approach simplifies the initial data extraction and interpretation; however, these baseline values are subsequently adjusted for each individual segment and crossover type to capture the specific material intensities required for a comprehensive market analysis. Utilizing both methods facilitates a robust assessment and provides a mechanism for cross-validation, ensuring a statistically reliable dataset across all drivetrain technologies.

### Modelling vehicle composition based on total material content (Top-down)

#### Basic procedure

**Fig. 3:**
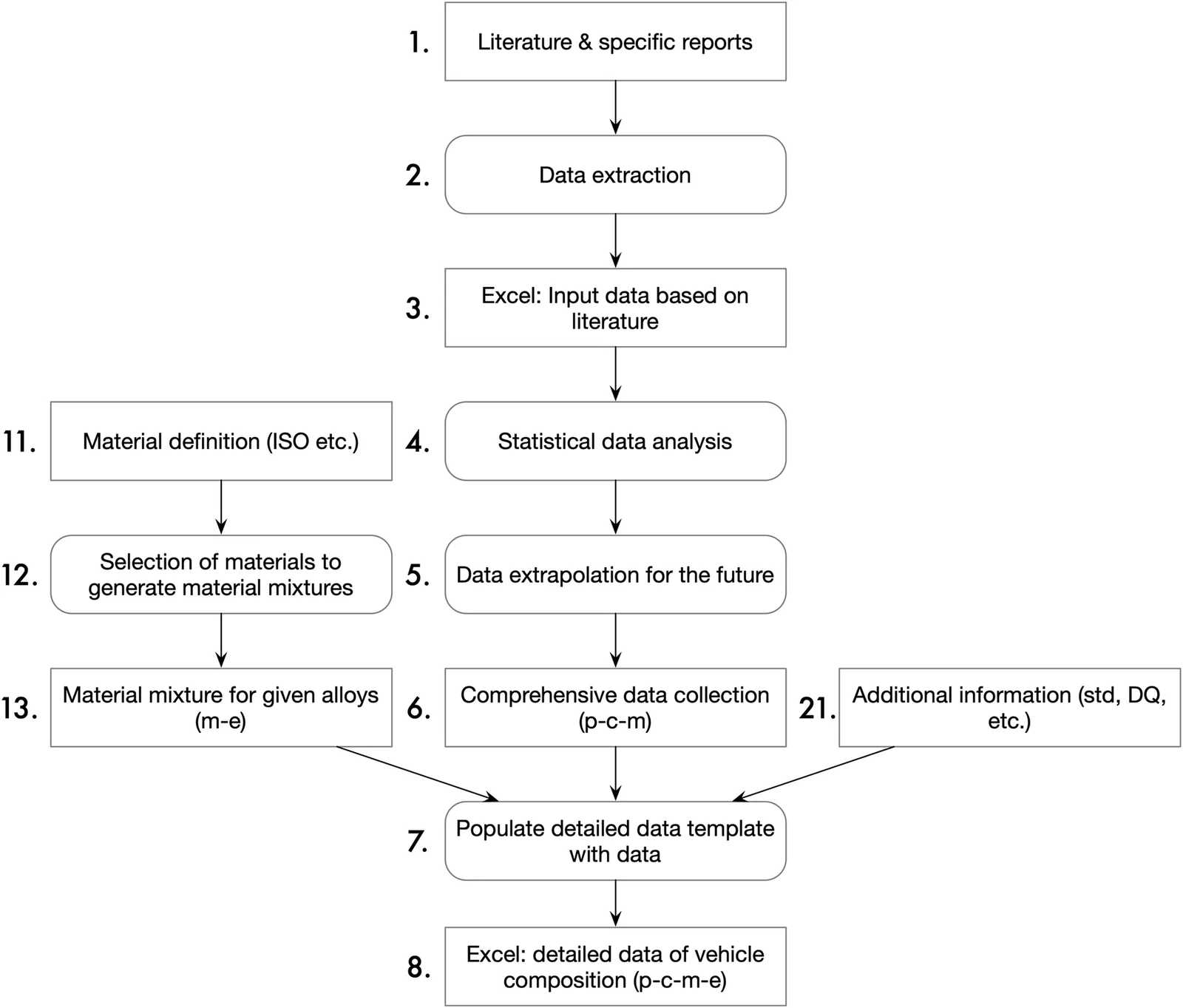
Workflow for compiling product, component, and material compositions from the overall metal alloy content of a vehicle (top-down approach).

The flowchart in Fig. 3 illustrates the methodological steps used to establish a robust dataset of vehicle material composition for the top-down approach. In step 1, literature, specific technical publications^33–35,37,60–69^, previous studies^51^, and reports were identified as the primary data sources. In step 2, relevant information from these sources was systematically extracted and structured into an input database, represented as step 3. Within this database (2_VehicleCompositionNewData.xlsx)^70^, each individual sheet collects the results of the data extraction process from the various sources. Their respective web addresses are given in the references, ensuring a structured and traceable repository for subsequent quantitative analysis.

In step 4, a statistical analysis (VehicleAlloysData.Rmd)^70–72^ was performed on all relevant and recent literature and report datasets, addressing aluminum alloys, steel alloys, electronics, and catalytic converters for ICEVs, HEVs, and PHEVs. The resulting statistical profiles were then consolidated with a pre-existing dataset from a previous study (1_JRCData_1980_2010.xlsx)^70^, which provided a less granular level of material detail, thereby extending the temporal and compositional coverage of the input data. A detailed description of the statistical procedures employed in this phase is provided in the following section. It resulted in the intermediate dataset (21_CompositionData.xlsx)^70^.

This subsequent analysis also encompassed step 5, which detailed the data extrapolation procedure and the specific adaptations required for different drivetrains, transitioning from the baseline ICEV to BEV, HEV, and PHEV architectures. This adaptation is executed via dedicated additional code (VehicleComponentELVHEVPHEVOther.Rmd)^70^ that utilizes the intermediate datasets (21_CompositionData.xlsx)^70^ generated by the initial procedure. This separation was intentional, as it allowed for the integration of specialized external sources necessary to accurately model the distinct material requirements of electrified drivetrains. This step generated a further intermediate dataset comprising all the relevant information from the product down to the material level (23_CompositionData.xlsx)^70^ shown as 6. step.

In step 7 (VehicleComostionDataset.Rmd)^70^, the material - element mixtures derived in step 13 (22_MaterialElementComposition.xlsx) are combined with the supplementary technical information collected in step 21 (13_VehicleCompositionAdditonalInformation.xlsx) to generate the detailed dataset of vehicle compositions (ELV_1980_2050.xlsx)^57^. This dataset covers all six drivetrain types (ICEV, BEV, HEV, PHEV, MHEV, FCEV), the twelve conventional vehicle segments (A–F), the crossover segments (JA–JF), and the standard car model. The resulting data structure integrates all four hierarchical descriptors - product, component (Level 0), material, and element (p-c-m-e) - ensuring a consistent representation of material flows across the complete vehicle spectrum.

The material-element mixtures themselves are constructed (VehicleMaterialMix.Rmd)^70^ using composition limits specified in international alloy standards (e.g., ISO, EN, ASTM) (14_VehicleMaterialElementDefinitions.xlsx)^70^ together with engineering knowledge of the typical alloys used within the relevant component families (22_MaterialElementComposition.xlsx)^70^. These mixtures are then mapped onto the component-level mass distributions to calculate the full elemental breakdown of each vehicle type. By consolidating standardized alloy chemistry with component-specific material deployment, this step produces a scientifically robust and fully traceable dataset of vehicle material and elemental composition suitable for further analysis and scenario modelling.

#### Application of the modelling procedure

##### Aluminum

The automotive industry represents one of the most resource-intensive sectors globally, with the European market alone accounting for approximately 42% of total aluminum demand, equivalent to roughly two million tons annually^59,73^. This high material intensity makes the sector a primary focus for stakeholders across the value chain, from primary producers to recyclers, who require precise data on current material demands and robust forecasts for future trends. To address this need, this study relied extensively on a series of strategic research reports published by Ducker over more than a decade (2012, 2016, 2019, and 2022)^10,33–35,37,61^. These reports served as a critical longitudinal data source, providing comprehensive analyses and estimates of automotive aluminum content in Europe. A central feature of these datasets was the description of a standard passenger car^34^, a composite model that aggregated various vehicle segments to represent the weighted average of aluminum intensity across the fleet. Recent editions of these studies^34,63^ had progressively expanded their granularity to explicitly include all electric vehicle segments, capturing the significant shift in aluminum utilization driven by the transition to electric mobility.

The individual data points spanning the duration of these multi-year reports were systematically collected and digitized into structured Excel Table (2_VehicleCompositionNewData.xlsx). These compiled datasets, which draw from periodic industry updates and complementary scientific literature, served as the empirical foundation for the top-down modeling of material intensities. To derive continuous trends from these discrete data points, statistical processing was conducted in R using the ggplot2 package^74^. Specifically, the geom_smooth() function was employed using the default Locally Estimated Scatterplot Smoothing (LOESS) method. This non-parametric approach was particularly suited for capturing the non-linear evolution of material content over time without imposing a rigid functional form on the data.

The quality of the statistical fit was consistently verified by plotting the raw data points against the smoothing curves produced by geom_smooth() and the LOESS fitting algorithm. In instances where the default fit did not sufficiently capture the underlying industry trends, the smoothing parameter (span) was manually adjusted to achieve a more accurate representation of the empirical data. Once a satisfactory smoothed trend was established for the standard car, the loess() function was applied to interpolate annual data points within the available timespan. This procedure ensured that the resulting annual datasets were grounded exclusively in the empirical time series and projections reported in the Ducker studies, providing a unified and scientifically robust baseline. These interpolated values were essential for the subsequent phase of the analysis, where the standard-car intensities were adapted to the specific material requirements of individual vehicle segments and diverse drivetrain architectures.

**Fig. 4:**
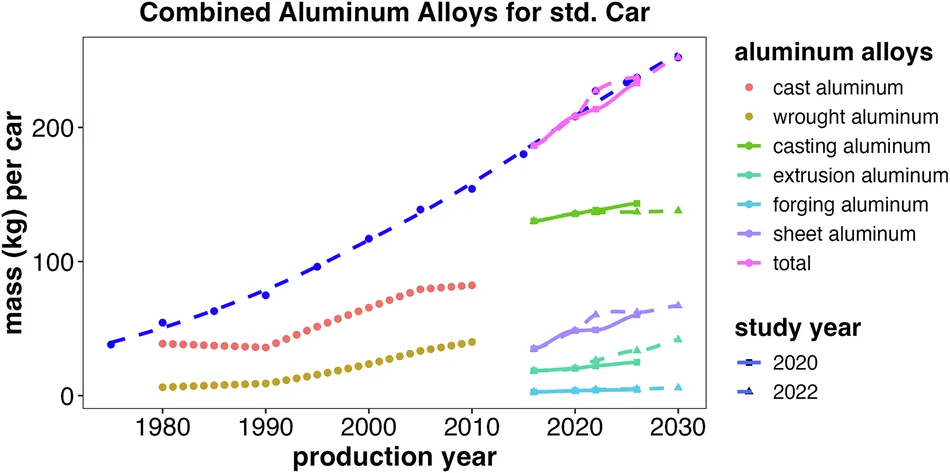
Historical alloy data (1980–2010) compared with recent studies. The 2020 and 2022 data points specifically originate from Ducker studies. The figure also includes the total aluminum alloy sums from these recent studies and the cumulative sums across all studies (blue dashed line).

Figure 4 provides a longitudinal overview of aluminum alloy utilization and predictions from 1980 to 2030^10,33,34,37,51,61^, integrating historical data with contemporary findings. The figure displays individual data points alongside aggregated totals (blue line) for all reported alloys, enabling a direct assessment of temporal trends and shifts in material composition. This comparative approach facilitates the evaluation of how alloy application has evolved in response to changing automotive engineering requirements.

The selection of wrought aluminum alloys is fundamentally driven by the required product form - sheet, extrusion, or forging - and the specific performance criteria of the component. Body sheets primarily utilize the 5xxx (Al-Mg) series for inner structures and battery housings due to superior formability and weldability, while the 6xxx (Al-Mg-Si) series dominates outer panels owing to its paint-bake hardening capabilities. Extruded components, such as space frames and crash boxes, rely almost exclusively on the 6xxx series for its excellent extrudability and corrosion resistance, with high-strength 7xxx (Al-Zn) alloys selectively applied in safety-critical structures. Forged parts, including wheels and suspension components, utilize both 6xxx and 7xxx series alloys to achieve the high fatigue resistance and mechanical strength required for load-bearing applications. Collectively, the 5xxx and 6xxx series form the structural backbone of the vehicle, while the 7xxx series is reserved for specialized, high-load, or crash-relevant functions.

**Fig. 5:**
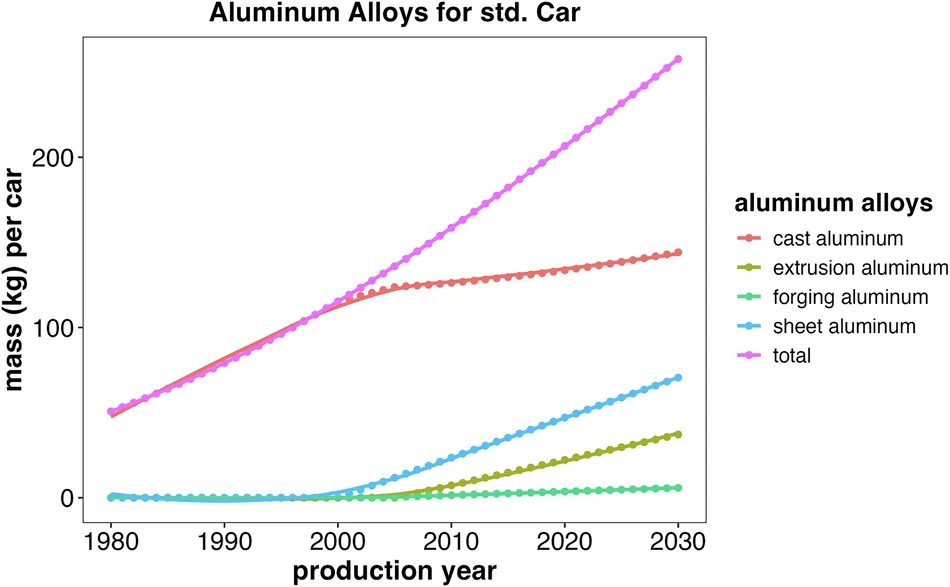
Interpolated annual data points for individual aluminum alloys, representing varying processing and performance requirements, are presented together with their aggregated sum.

Figure 5 shows the interpolated annual data points for the main aluminum alloy categories, together with their aggregated total, providing a continuous temporal profile of alloy use in automotive manufacturing. Smoothed trend lines were generated using the geom_smooth() function in the R^72^ package ggplot2^74^, applying LOESS interpolation to capture the underlying long-term trajectories. The resulting curves illustrate distinct shifts in material strategies: aluminum sheet and forging alloys exhibit a consistent increase over time, while cast aluminum shows a reduced increase. These developments reflect a broader industry transition toward higher-performance wrought products, driven by evolving structural requirements and improvements in forming and joining technologies.

##### Iron & Steel:

**Fig. 6:**
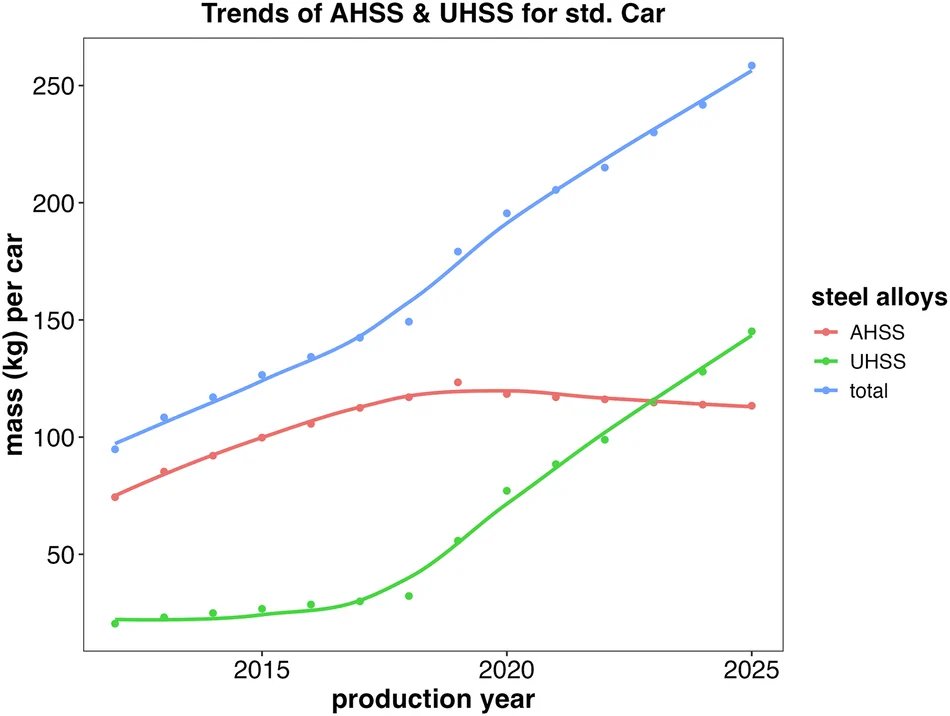
Temporal trends of Advanced High-Strength Steel (AHSS) and Ultra High-Strength Steel (UHSS), highlighting the progressive replacement of AHSS by UHSS over time.

As shown in Fig. 6, the temporal evolution of the newest steel classes - Advanced High-Strength Steels (AHSS) and Ultra-High-Strength Steels (UHSS)^64,65,67,75–77^ - demonstrates a clear shift in automotive material strategies. While AHSS initially dominated the high-strength segment, UHSS exhibits a continuous and steady increase, progressively replacing AHSS in many structural applications. This development reflects improvements in metallurgical design, forming technologies, and crash-performance requirements, which increasingly favor steels with higher yield strength, enhanced energy absorption, and reduced component weight. As a result, UHSS has become an essential material for modern body-in-white architectures, particularly in safety-critical zones.

**Fig. 7:**
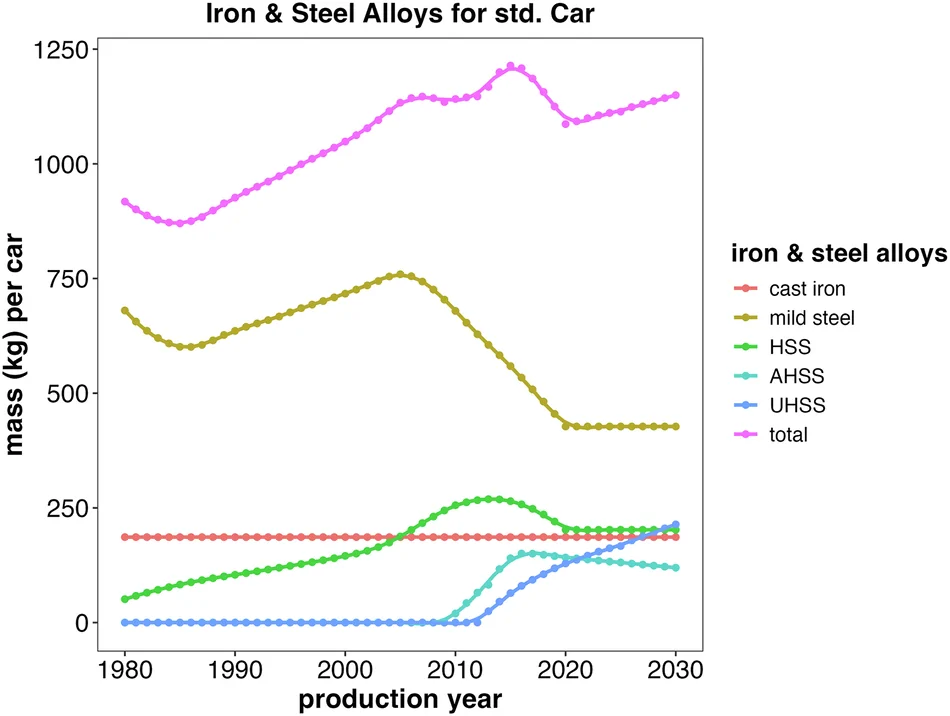
Temporal evolution of cast iron and different steel alloys in automotive production, illustrating the gradual decline of mild steel alongside the increasing adoption and diversification of advanced steel grades.

Figure 7 shows the temporal evolution of iron- and steel-based alloys in automotive production. The introduction of Advanced High-Strength Steels (AHSS) and Ultra High-Strength Steels (UHSS) had significantly reshaped earlier patterns, constraining the growth of conventional High-Strength Steel (HSS) and accelerating the decline of mild steel. This shift mirrors the trends observed for aluminum in Fig. 5, where increasing use of higher-performance wrought alloys similarly reflects industry-wide demands for improved crash performance, weight reduction, and structural efficiency. In addition, magnesium alloys had been introduced as a further lightweighting measure, selectively replacing aluminum components in premium vehicle segments (E and F), though their adoption remained limited to specific models rather than representing a broad industry trend. While this development is expected to continue, insufficient data currently exist to determine whether magnesium adoption will accelerate or extend to mid-range segments (C and D) or entry-level vehicles (A and B). However, the modelling of steel and iron alloys is less robust than for aluminum due to the smaller number of studies and available data points, which limits the resolution and precision of the derived trends^63,64,68,78–80^.

##### Electronics

**Fig. 8:**
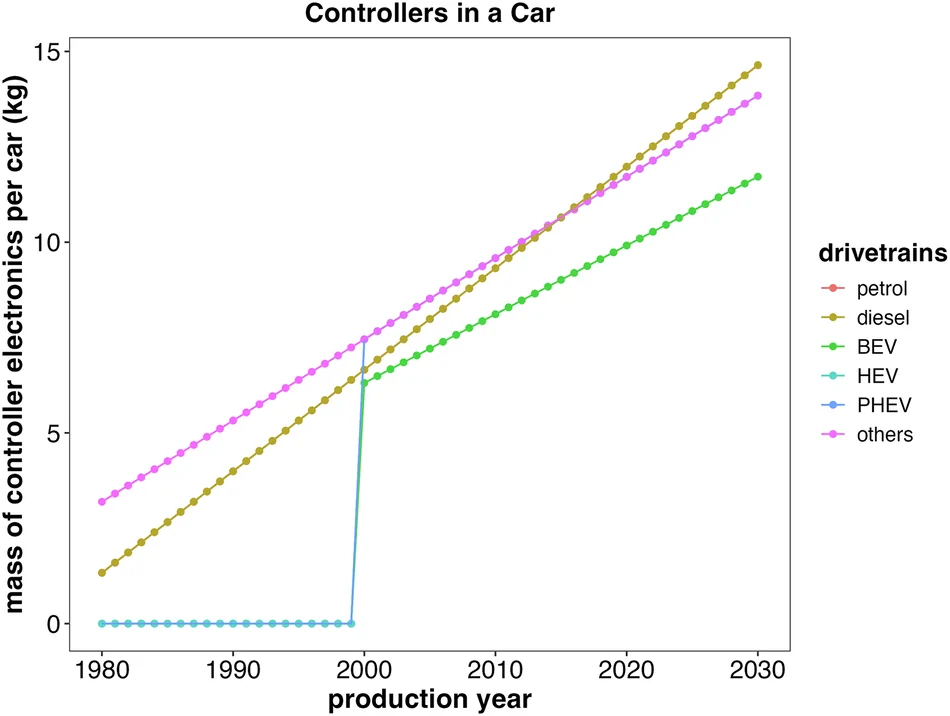
Temporal evolution of the approximate mass of electronic control units (ECU) across different drivetrain types. As essential components, control units manage drivetrain operation as well as broader vehicle electronic functions. Data for BEV, HEV, and PHEV are only available from 2000 onwards, corresponding to their market introduction.

Figure 8 presents the estimated per-vehicle mass of drivetrain-specific electronic control units (ECUs) for internal-combustion (ICEV), hybrid (HEV/PHEV), and battery-electric (BEV) configurations. The analysis is based on the EVA2 study^81,82^, which characterized electronic components from end-of-life ICEVs, supplemented by component catalogues for model years 2000–2022. Linear regression is applied to highlight long-term trends, capturing the pronounced growth in electronic content following the market introduction of electrified drivetrains. For ICEVs, the mass curve primarily reflects the engine and transmission control units, while for BEVs and PHEVs, comparable supervisory functions are increasingly integrated into traction inverters and high-voltage domain controllers.

It is important to note that this mass-based modeling approach, derived from legacy recycling data, may not fully capture the rapid transition toward software-defined vehicles (SDVs). In modern BEV architectures, controllers are becoming more centralized and computationally intensive, shifting the focus from hardware mass to the complexity and surface area of Printed Circuit Boards (PCBs). Furthermore, this study does not account for the additional hardware requirements of Level 5 autonomous driving. While a transition to complexity-based modeling is recognized as more representative of future trends, such a shift remains outside the current scope. Consequently, while the data illustrates a steep increase in ECU mass for BEVs, the results likely underestimate the total electronic value and functional density of next-generation electric platforms^26,83^.

#### Uncertainty, data variability and data quality

The dataset developed in this study provides estimated average material and elemental compositions for six drivetrain types across six vehicle segments and their corresponding crossover variants, as well as an aggregated “standard car”^34^. However, it is important to emphasize that these values represent segment-level averages. In practice, both material use and alloy selection differ substantially between segments and even more so between individual vehicle models produced by different manufacturers.

The reports and studies used as primary data sources do not provide uncertainty information^84–86^ for their material or alloy estimates, nor do they document how the underlying sample of vehicles was analyzed. Although these publications often aggregate data from dozens of individual car models, the absence of methodological transparency makes it impossible to derive statistically meaningful variability directly from the reported material values.

To address this limitation, variability was approximated using the range of curb weights observed for different car models within the same segment. While weight variation reflects differences in design, materials, and manufacturing strategies, it does not capture the full variability of alloy use or elemental composition. As such, this method provides only a coarse indicator of heterogeneity. Recognizing these limitations, the present study applies a data-quality–based uncertainty framework to derive reproducible uncertainty estimates^87^.

This framework evaluates each data source along established dimensions of data quality - accuracy, completeness, consistency, validity, and timeliness - and assigns one of four reliability levels. These qualitative ratings are translated into quantitative uncertainty ranges (e.g., ±10%, ±20%, ±40%, ±60%), enabling a consistent and transparent propagation of uncertainty throughout the analysis. The resulting uncertainty intervals therefore reflect the strength and reliability of the evidence, rather than statistical variance in a strict probabilistic sense.

It must be clearly acknowledged that uncertainties derived in this way are themselves uncertain. They should not be interpreted as precise confidence bounds; instead, they represent indicative ranges that convey the level of confidence appropriate for segment-level, literature-based material composition data. By combining limited empirical variability with a structured data-quality assessment, this study provides a scientifically defensible and reproducible approach to quantifying uncertainty in vehicle material and elemental composition under conditions of incomplete information.

#### Dataset verification and mass balance

Verification of a high-resolution material dataset presents significant challenges, as the values represent segment-level averages rather than the specifications of a single vehicle model. Because material strategies vary between manufacturers even within the same production year, direct comparisons with individual vehicles are of limited utility for validating the entire dataset. Instead, verification is achieved through a systematic mass-balance approach, comparing the aggregated mass of all modeled materials against known historical trends of total vehicle curb weight^86,88–91^.

This method relies on the principle that the sum of all component families must equal the total vehicle mass. While this study focuses on metallic alloys and critical materials, the non-metallic fraction - comprising plastics, glass, fluids, and elastomers (categorized as “rest”) - is accounted for using established industry weight percentages. By integrating these fractions, the total calculated mass can be benchmarked against official manufacturer specifications.

**Fig. 9:**
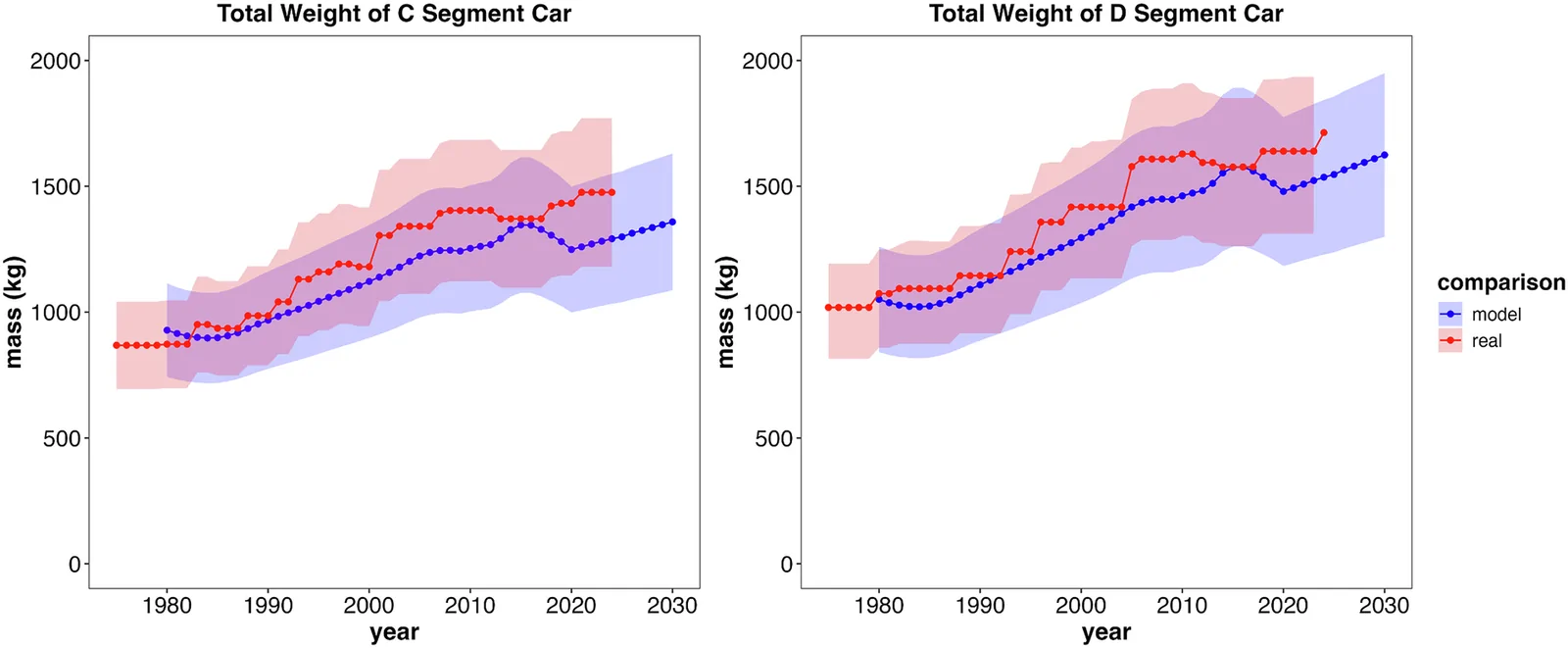
Comparison of published vehicle masses for C- and D-segment cars (various manufacturers) with total masses modelled from reported material compositions. The blue line represents the modelled composition data, and the red line represents the weight of selected C- and D-segment cars. The strong agreement is expected, as these segments are similar to the standard car forming the dataset’s foundation.

As demonstrated in Fig. 9, the total vehicle masses derived from the material-composition model for the C- and D-segments show a strong correlation with reported curb weights^42^ from major manufacturers^40,92^. This high degree of correspondence is expected, as these mid-sized segments closely align with the “standard car” architecture used as the primary reference for the top-down analysis. However, it must be noted that this longitudinal verification is primarily applicable to Internal Combustion Engine Vehicles (ICEVs), where decades of empirical data allow for the establishment of robust historical trends.

For HEV, PHEV, and BEV architectures, a similar historical trend analysis is not yet possible due to the lack of long-term longitudinal data. In these cases, verification relies on a cross-methodological validation between the top-down alloy intensities and the bottom-up component-based aggregation. By ensuring that the sum of individual electrified components (e.g., traction motors, power electronics, and battery housings) aligns with the total material demand predicted by the top-down model, the study provides a mutually validating framework. This dual-track approach ensures that the resulting material and elemental profiles are internally consistent and physically representative of the rapidly evolving European automotive fleet.

### Modelling vehicle composition based on component families (Botton-up)

**Fig. 10:**
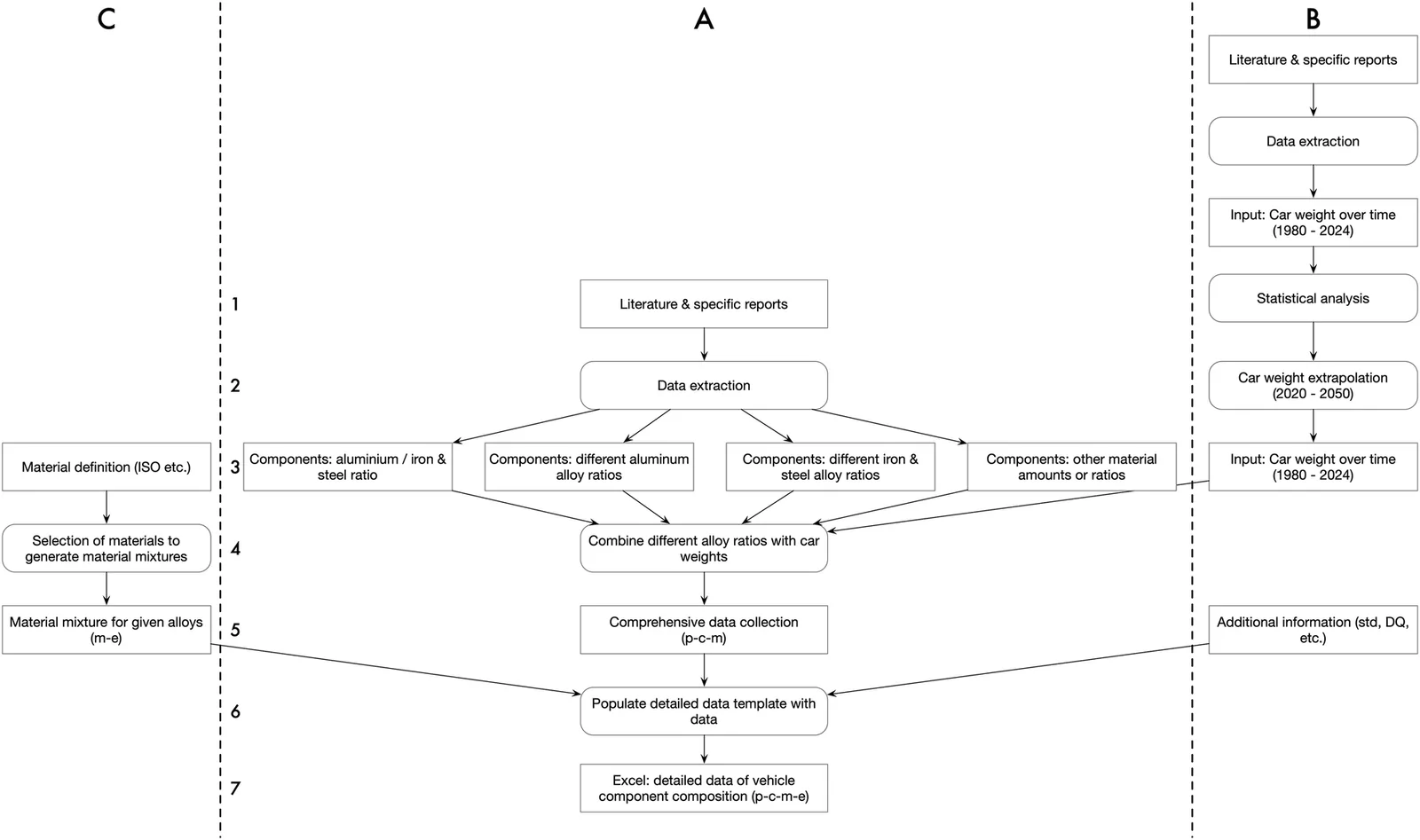
Workflow outlining the procedure for determining the detailed composition of all major automotive components.

The bottom-up approach provides a granular decomposition of vehicle architecture^77,80,93–95^, enabling the systematic tracking of material concentrations within specific component families. This methodology is illustrated in the tripartite flowchart in Fig. 10, which distinguishes between the core analytical sequence (**Part A**), the integration of vehicle mass dynamics (**Part B**), and the standardization of elemental material composition (**Part C**).

#### Core Analytical Sequence (Part A)

The central process began with the extraction of high-resolution data from contemporary industry reports and technical literature (Steps 1–2). In Step 3, this information was structured into four specialized relational databases that defined the material distribution at the component level (Level 1, as defined in Table 3) (Data/31_ComponentMaterial.xlsx)^70^:

- **Aluminum-to-Steel Ratios:** Established the primary structural competition between ferrous and aluminum solutions for each component family (e.g., BIW, closures, chassis) (componentCarSegment).
- **Aluminum Alloy Ratios:** Provided a fine-grained distribution of specific wrought and cast aluminum series within each component (componentAluminum, componentMagnesium).
- **Ferrous Alloy Ratios:** Defined the distribution of steel grades, from mild steel to UHSS, across the vehicle architecture. (componentSteel).
- **Other Materials and Battery Mass:** Accounted for non-metallic fractions and the significant mass contribution of battery packs in HEV, PHEV, and BEV drivetrains. More detailed information concerning the battery composition is provided in the next section (componentCarRatio).

In Step 4, these ratios were synthesized (VehicleComponentFamilyComonentData.RmD)^70^ with the segment-specific vehicle weights (Data/7_CarWeight.xlsx) derived from Part B. This integration generated a comprehensive material inventory (Step 5) that establishes the product-component-material (p-c-m) relationship (Data/35_VehicleComponentData.xlsx)^70^.

#### Integration of mass and elemental data (Parts B and C)

To ensure the model reflected physical reality, Part B incorporated longitudinal curb weight data (1980–2024) and future projections (2025–2050). This allowed the percentage-based material ratios to be converted into absolute mass values (kg) for each vehicle segment. Simultaneously, Part C provided the elemental profiles (m-e) for each defined alloy, utilizing the same international standards (ISO, ASTM)^54–56,96–98^ employed in the top-down approach.

In Step 6, these disparate data streams (VehicleComonentDataset.RmD)^70^ were populated into a unified template. By mapping the elemental chemistry from Part C (Data/34_MaterialElementComponentComposition.xlsx)^70^ onto the mass-balanced component profiles from Part A, the final dataset (Step 7) (ComponentData/ELVComponent_1990_2050.xlsx)^57^ achieved a full p-c-m-e resolution.

#### Functional significance

This hierarchical structure is essential for identifying “material hotspots” - specific components where critical or strategic raw materials (CRMs/SRMs) are concentrated. For example, while smaller, cost-sensitive segments (A and B) maintain a higher reliance on ferrous alloys, larger segments (E and F) and crossovers (J-segment) increasingly utilize aluminum and magnesium to offset the mass of advanced drivetrains and premium equipment. By normalizing alloy distributions as percentage ratios that sum to 100% within each material group, the methodology ensures a consistent and transparent mass balance across all 12 vehicle segments and six drivetrain configurations^34,37,60,63^. This level of detail is critical for informing targeted recovery strategies in end-of-life vehicle recycling flows.

**Fig. 11:**
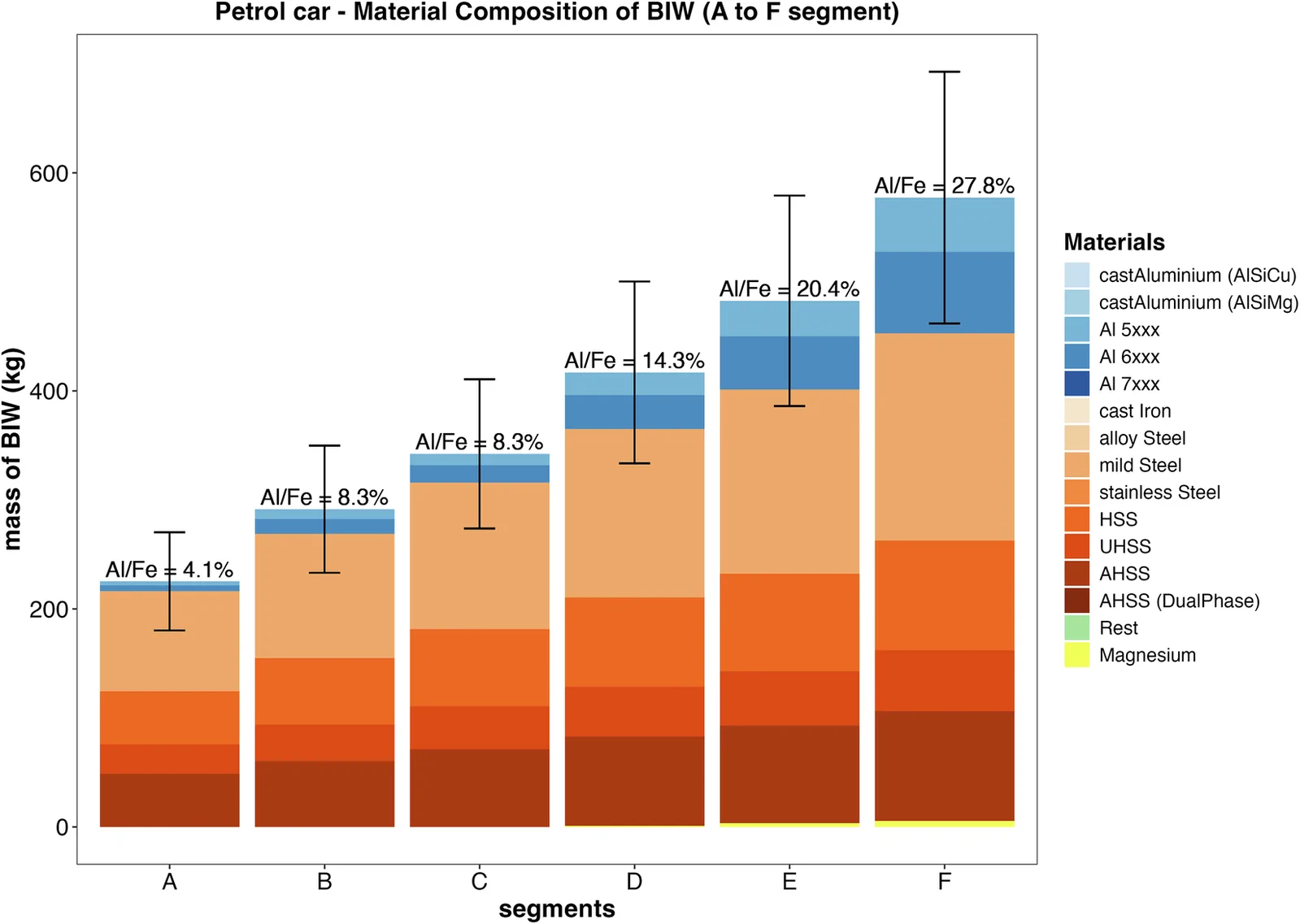
Variation in aluminum versus iron and steel usage across car segments (A to F). The data illustrate a clear trend towards increased lightweight material adoption, particularly aluminum, correlating with reduced cost sensitivity in larger, more premium vehicle segments.

**Fig. 12:**
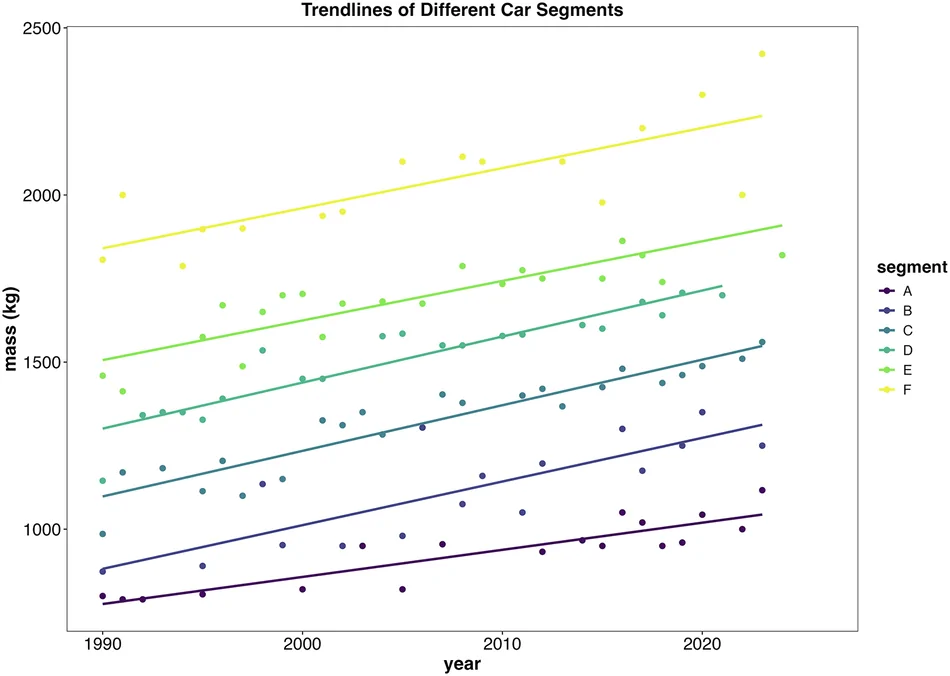
Increase in vehicle mass by segment, 1990–2024. Except for the smallest (Segment A), all segments show near-uniform annual gains of ≈11–14 kg/yr^42^.

The percentage ratios established in the bottom-up framework provide the mathematical basis for calculating the proportional contribution of each alloy within a specific component family. When these ratios are applied to the average curb weight of a defined vehicle segment and model year, they enable the estimation of the absolute mass (kg) for every alloy across the vehicle architecture. Aggregating these values across all component families yields the total material mass of the vehicle, a process that serves as a critical internal validation of the percentage allocations (see Fig. 11). This systematic procedure ensures a comprehensive and mass-balanced assessment of material intensity across all twelve vehicle segments and six drivetrain configurations (see Fig. 12).

A recognized limitation of this methodology is that the component-level percentage ratios are currently static and do not dynamically reflect longitudinal shifts in base material composition—such as the substitution of mild steels with AHSS/UHSS or the replacement of ferrous components with aluminum and magnesium. While these trends could be integrated using the top-down material-based data described previously, such a step was intentionally avoided to preserve the analytical independence of the two methodologies. Maintaining this separation is essential for the objective cross-verification and validation of results between the top-down and bottom-up approaches. Furthermore, within the immediate temporal scope of this study, the impact of these material shifts is considered secondary to the inherent data uncertainties (estimated at 20–30%). By prioritizing methodological independence, the study ensures that the convergence of results between the two models provides a robust and scientifically defensible characterization of automotive material flows.

#### Essential batterie composition

The transition to electric mobility has necessitated a detailed characterization of traction battery compositions, as these assemblies represent a significant portion of the total vehicle mass and contain critical raw materials (CRMs). This study focuses on Lithium-Nickel-Manganese-Cobalt (NMC) and Lithium Iron Phosphate (LFP) chemistries, which currently represent the dominant technologies in the Battery Electric Vehicle (BEV), Plug-in Hybrid (PHEV), and Hybrid Electric Vehicle (HEV) markets.

To capture the technological diversity within these categories, the dataset was distinguished between various NMC formulations based on their cobalt intensity:

- **NMC Low:** Primarily NMC333 architectures.
- **NMC Medium:** Focuses on NMC532 and NMC622, including subtypes utilizing silicon-graphite anodes.
- **NMC High:** Represents high-nickel formulations such as NMC811 and NMC955, which are increasingly favored for their higher energy density and reduced cobalt dependency.

The elemental and material composition data for these batteries were generated using the BatPaC (Battery Manufacturing Cost Estimation) simulation software developed by Argonne National Laboratory^99^. This tool enabled a precise breakdown of active materials, current collectors, electrolytes, and structural pack components (e.g., enclosures and thermal management systems). While sodium-ion and solid-state batteries represent emerging technologies, they were excluded from the current scope due to their limited market penetration as of early 2026 and the lack of standardized industrial composition data.

**Fig. 13:**
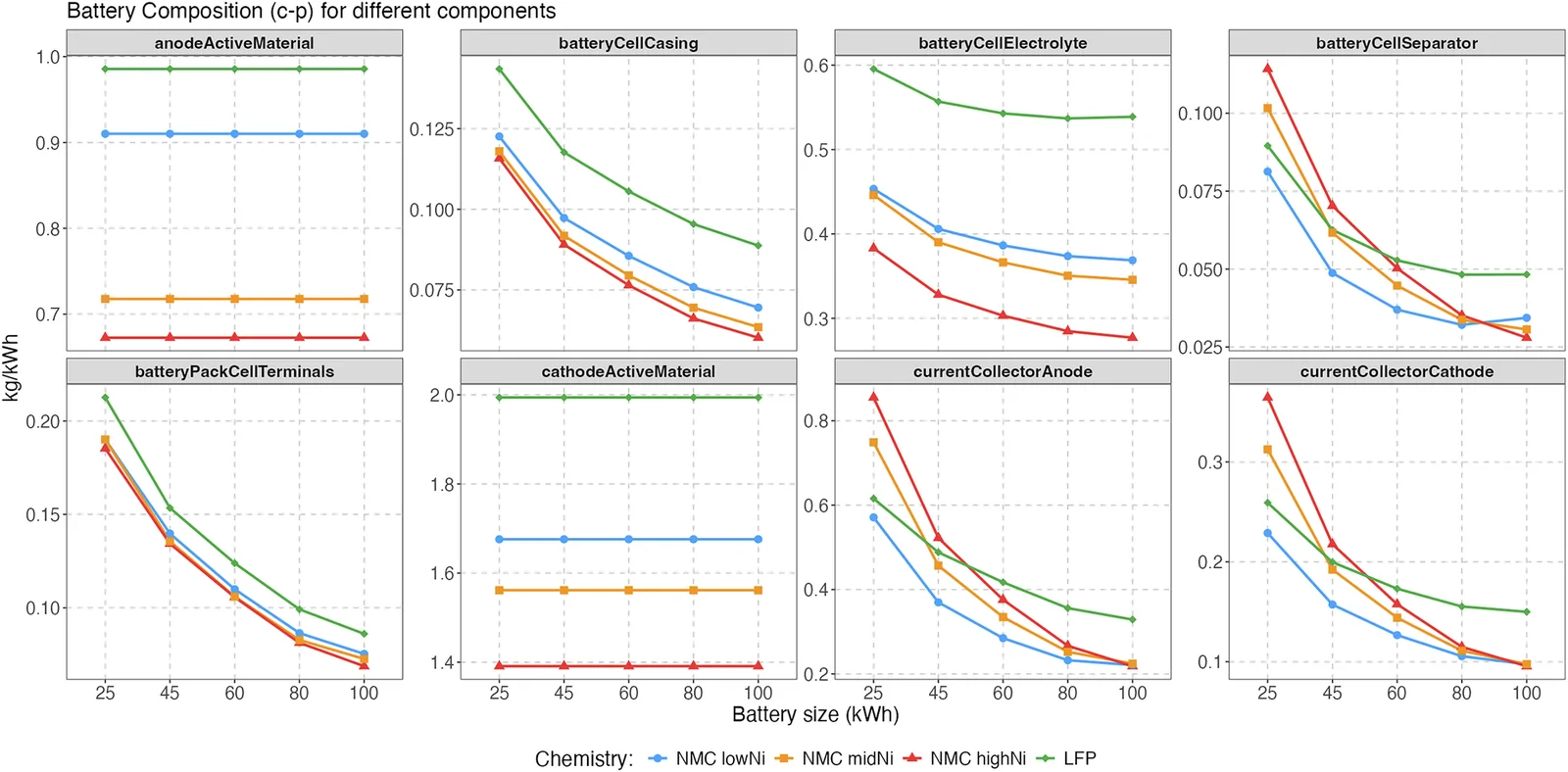
BEV-battery composition on component level for low, medium and high nickel content NMC and LFP batteries. The amounts are given for different battery sizes (kWh) and weight per kWh (kg/kWh).

The battery composition data (BATT_consolidated_composition.xlsx)^57^ was integrated into the broader vehicle material dataset by calculating the absolute mass contribution for each vehicle segment. This is achieved by multiplying the chemistry-specific material intensities (kg/kWh), derived from the BatPaC simulations, by the average battery capacities (kWh) assigned to each segment and drivetrain type (Fig. 13). This methodology ensured that the higher curb weights of electrified vehicles were accurately reflected and grounded in the specific chemical requirements of their respective energy storage systems. The resulting battery data is provided as a standalone dataset to facilitate targeted analysis of battery-specific material flows and recycling potentials.

## Data Records

The data record structure employed in this study was developed within the framework of the FutuRaM project to ensure a standardized, high-resolution approach to material flow analysis. The dataset follows the^100^ – Product-centric Approach, which is publicly available on Zenodo [https://doi.org/10.5281/zenodo.17176540]^101^ To ensure full interoperability and transparency of the data, all entries are mapped to the FutuRaM Code List Repository [https://doi.org/10.5281/zenodo.15681409]^102^, which provides the essential nomenclature and classification codes for products, components, materials, and elements (p-c-m-e).

The following tables and short explanation detail the data architecture, categorized into input, intermediate, and final datasets. These records represent the execution of the methodology described in Methods, processed through a custom R-based analytical pipeline. The input datasets comprise the raw historical and contemporary vehicle composition data, while the intermediate datasets represent the results of the statistical harmonization and LOESS^74^ interpolation procedures. The final datasets provide the consolidated, drivetrain-specific material and elemental intensities for the European passenger car fleet from 1980 to 2050. A comprehensive list of the R^72^ scripts and computational modules used to generate these records is provided in the subsequent tables to facilitate reproducibility.

The data architecture is organized into three distinct stages, reflecting the progression from raw data ingestion to the final synthesized outputs. Table 4 lists the primary input datasets, which include historical car weights, segment-specific mass developments, and raw material composition data sourced from literature and previous JRC studies^51^. These files provide the foundational “product-centric” and “component-centric” keys required for the initial mapping. Table 5 details the intermediate datasets generated computationally through the R-based pipeline. These records represent the results of the statistical harmonization, LOESS interpolation, and the integration of drivetrain-specific adaptations for electrified vehicles (BEV, HEV, PHEV, and FCEV). They are all located in the folder Data. Finally, Table 6 presents the consolidated datasets, which provide the high-resolution material and elemental intensities (p-c-m-e) for the European passenger car fleet across the entire 1980–2050 temporal scope.

**Table 4:** Overview of input data collections used to generate the final vehicle composition dataset, including their temporal coverage, geographical scope, data type (top-down or bottom-up), and primary role in the harmonization and interpolation workflow. (https://doi.org/10.5281/zenodo.17894289)^70^.

Filename	Description
0_CarSegments	Weight development of different car segments
1_CarWeights	Car weights & product keys
1_JRCData_1980_2010	Data from previous study used from 1980 to 2010
2_VehicleCompositionNewData	Literature data for all the different alloys and materials etc.
3_VehicleCompositionPredictions	Literature data used for future development (20230)
5_VehicleComponentCompositionNewData	Data for component composition based on literature
7_CarWeights	Car weights used for each year
12_VehicleCompositionDataBase	Product, component, material keys used for total vehicle composition dataset
13_VehicleCompositionAdditionalInformation	Additional information used for total vehicle composition dataset
14_VehicleMaterialElementDefinitions	Specification of the standard material mixtures for total vehicle composition
15_VehicleComonentMaterialElementDefinitions	Actual standard alloy specifications
31_ComponentMaterial	Specification of the standard material mixtures for component vehicle composition
32_VehicleComponentDataBase	Product, component, material keys used for component vehicle composition dataset
33_VehicleComponentAdditionalInformation	Additional information used for component vehicle composition dataset

**Table 5:** Overview of intermediate data collections generated computationally during the processing pipeline, including their temporal coverage, and primary role in the harmonization and interpolation workflow. (https://doi.org/10.5281/zenodo.17894289)^70^.

Filename	Description
21_CompositionData	Total vehicle composition p-c-m dataset
22_MaterialElementComposition	Total vehicle composition m-e dataset
23_CompositionDataUpdated	Updated total vehicle composition p-c-m dataset for special cases of BEV, HEV, PHEV and FCEV
34_MaterialElementComponentComposition	Component vehicle composition m-e dataset
35_VehicleComponentData	Component vehicle composition p-c-m dataset

**Table 6:** Overview of the final consolidated datasets providing high-resolution material and elemental intensities (p-c-m-e) for the European passenger car fleet, representing the final output of the analytical pipeline for the 1980–2050 temporal scope. (https://doi.org/10.5281/zenodo.17349758)^57^.

Filename	Description
ELV_1980_2050	Final dataset p-c-m-e of complete vehicle composition using large template
ELV_1980_2050_short	Final dataset p-c-m-e of complete vehicle composition using reduced template
BATT_consolidated_composition	Battery dataset for BEV, HEV and PHEV based on BatPac simulations
ELV_1980_2050_BEV.csv	Final dataset p-c-m-e of BEV vehicle composition in.csv format
ELV_1980_2050_Diesel.csv	Final dataset p-c-m-e of Diesel vehicle composition in.csv format
ELV_1980_2050_HEV.csv	Final dataset p-c-m-e of HEV vehicle composition in.csv format
ELV_1980_2050_Others.csv	Final dataset p-c-m-e of Others vehicle composition in.csv format
ELV_1980_2050_Petrol.csv	Final dataset p-c-m-e of Petrol vehicle composition in.csv format
ELV_1980_2050_PHEV.csv	Final dataset p-c-m-e of PHEV vehicle composition in.csv format
ELVComponent_1990_2050	Final dataset p-c-m-e of component vehicle composition using large template
ELVComponent_1990_2050_short	Final dataset p-c-m-e of component vehicle composition using reduced template
ELVComponent_1990_2050_BEV.csv	Final dataset p-c-m-e of BEV component vehicle composition in.csv format
ELVComponent_1990_2050_Diesel.csv	Final dataset p-c-m-e of Diesel component vehicle composition in.csv format
ELVComponent_1990_2050_HEV.csv	Final dataset p-c-m-e of HEV component vehicle composition in.csv format
ELVComponent_1990_2050_Others.csv	Final dataset p-c-m-e of Others component vehicle composition in.csv format
ELVComponent_1990_2050_PHEV.csv	Final dataset p-c-m-e of PHEV component vehicle composition in .csv format

The final datasets derived from the top-down approach are stored in the CompositionData folder, while all files generated through the component-based bottom-up procedure are in the ComponentData folder.

## Technical Validation

The technical integrity of the generated dataset is validated through a multi-step process involving cross-methodological comparison, historical benchmarking, and mass-balance closure.

### Cross-methodological consistency and statistical reliability

**Fig. 14:**
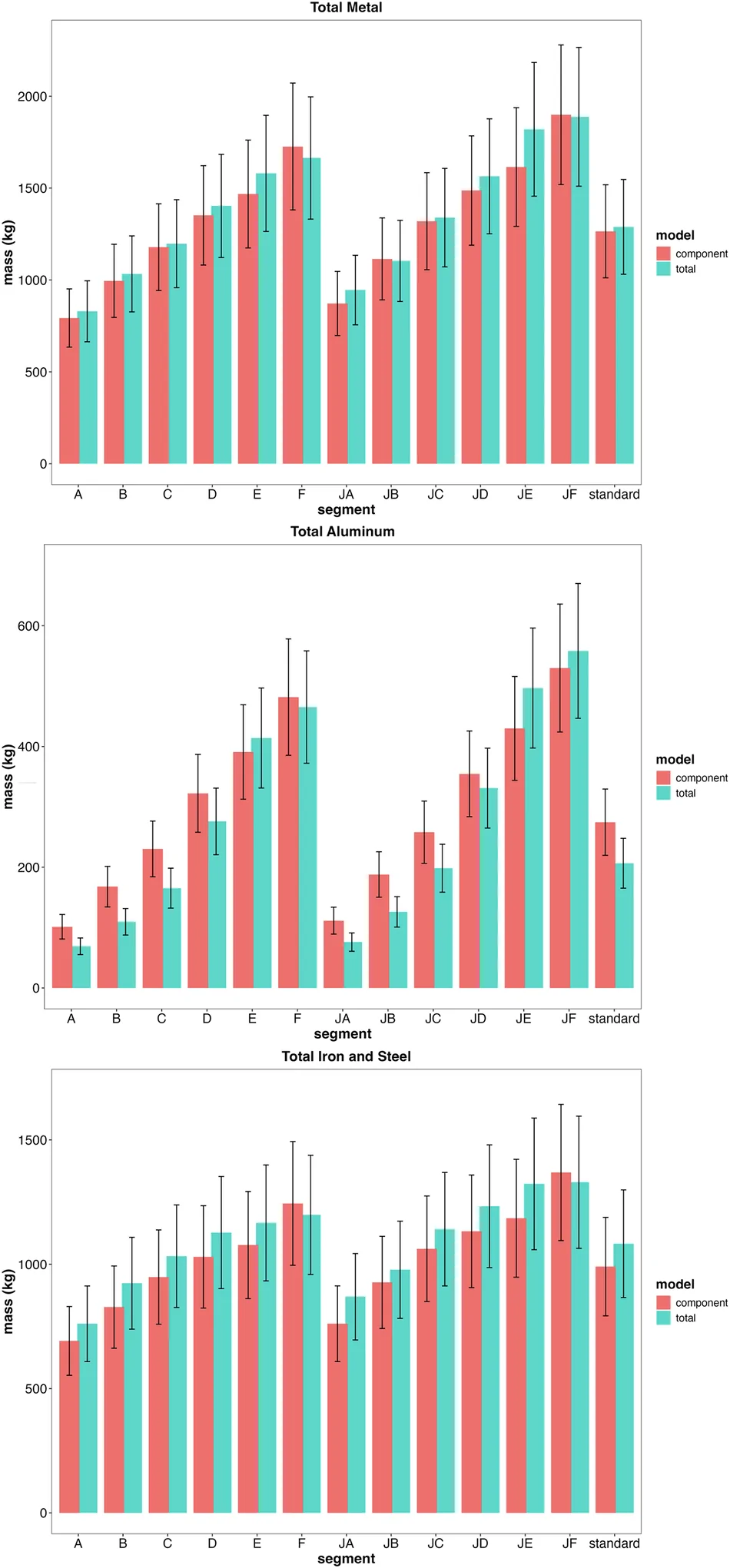
Cross-methodological validation of material mass intensities for petrol passenger cars (reference year 2020). The panels compare results from the top-down overall approach (Fig. 3) and the bottom-up component-level approach (Fig. 10) for: (**A**) total metal content, (**B**) aluminum, and (**C**) iron/steel. Error bars represent a ± 20% uncertainty range, corresponding to Data Quality (DQ) level 2. Pearson correlation coefficients (${\rm{r}}$) for the two methods are 0.980, 0.983, and 0.962 for panels A, B, and C, respectively.

The convergence of the two independent modeling paths - the top-down mass-balance and the bottom-up component-centric resolution - serves as a primary indicator of internal consistency. Figure 14 illustrates this comparison for the reference year 2020, showing the total mass of metals, aluminum, and iron/steel across different vehicle segments.

Statistical analysis confirms a high degree of correlation between the two methods. The Pearson correlation coefficient^103–105^ (r) for total metal content is 0.980, while aluminum and iron/steel show coefficients of 0.983 and 0.962, respectively^72^. These values confirm that the component-level distributions accurately aggregate to the total vehicle masses reported in industry literature. Discrepancies remain within the defined uncertainty range of ±20%, which corresponds to a Data Quality (DQ) level 2.

It should be noted that the original modeling was anchored to a “standard car” configuration, which primarily reflects a mixture of the C and D segments^42^. Consequently, the largest deviations are observed in the extreme segments, such as the small A-segment or the luxury F and SUV (JA/JF) segments, where specialized structural requirements or optional equipment deviate most significantly from the standard reference architecture.

### Mass-balance closure and historical benchmarking

The longitudinal accuracy and mass-balance closure of the model are verified by comparing the total modeled vehicle masses against reported curb weights from major manufacturers. As demonstrated in Fig. 9, the total vehicle masses derived from the material-composition model for the C- and D-segments show a strong correlation with empirical data. This high degree of correspondence is expected, as these mid-sized segments closely align with the “standard car” architecture used as the primary reference for the top-down analysis^34,37^.

However, it must be noted that this longitudinal verification is primarily applicable to Internal Combustion Engine Vehicles (ICEVs), where decades of empirical data allow for the establishment of robust historical trends. For newer electrified drivetrains, the validation relies more heavily on the cross-methodological alignment described in previous section.

### Sensitivity to drivetrain electrification

A specific validation challenge arises from the rapid transition to electrified drivetrains (BEV, PHEV, HEV). Technical validation in this context involves assessing whether the model correctly accounts for the additional cooling and structural requirements of electric power electronics. Although current data for these segments is less abundant than for ICEVs, the model’s integration of specialized “hotspot” components - such as inverters and battery casings - ensures that the resulting material intensities reflect known engineering requirements, such as the increased demand for high-conductivity aluminum alloys^10,34,36,37,63^.

## Usage Notes

The dataset is structured to support both aggregate and high-resolution material flow analyses across the European passenger car fleet. Data are organized by six primary drivetrain types - petrol, diesel, battery electric vehicle (BEV), hybrid electric vehicle (HEV), plug-in hybrid electric vehicle (PHEV), and Others - with annual resolution. While internal combustion engine drivetrains (petrol and diesel) are covered from 1980 to 2050, electrified drivetrains are included from their market introduction around the year 2000.

Within each drivetrain-specific record, information is provided for 13 distinct car segments. The data follows a hierarchical product–component–material–element (p-c-m-e) logic, where each row describes either the entire vehicle, a specific component family, the material composition of a component, or the elemental chemistry of a material. This structure allows users to flexibly aggregate data at different levels of granularity. It should be noted that the listed alloys represent statistically representative averages for the “standard car” within a given segment^34,37^; individual vehicle compositions may deviate from these values due to manufacturer-specific designs or optional equipment. All reported weights are approximate and should be used in conjunction with the associated uncertainty ranges (typically ±20%, corresponding to DQ level 2).

The complete dataset is openly available on Zenodo as part of the FutuRaM project: “FutuRaM – Composition – Consolidated Data – Vehicles” [https://doi.org/10.5281/zenodo.17349758]^57^. The repository includes:

1. Drivetrain-specific .csv files for overall vehicle composition (1980–2050) and component-resolved composition (1990–2050), named according to the format ELV[Component]_1990_2050_[Drivetrain].csv^57^.
2. Comprehensive Excel workbooks (ELV_1980_2050.xlsx and ELVComponent_1990_2050.xlsx)^57^ containing individual sheets for each drivetrain type and summary versions for convenience.

Due to the high resolution of the data, the total volume reaches approximately 10 GB across several hundred thousand records. Users should be aware that opening the full Excel workbooks or the largest.csv files may exceed the memory capacity of standard notebook computers. For efficient data handling, it is highly recommended to access and filter the dataset using scripting environments such as R or Python^71^. The associated FutuRaM Code List Repository [https://doi.org/10.5281/zenodo.15681409]^102^ provides the necessary metadata to interpret the categorical codes and variable names used throughout the files.

## Data Availability

The dataset can be accessed at https://doi.org/10.5281/zenodo.17349759.

## Code availability

The procedures used to compile the vehicle composition datasets are summarized in the flow charts in Figs. 3 and 10^70^. Rounded boxes denote manual steps (e.g. extraction and consolidation of literature data), whereas rectangular boxes represent input or intermediate Excel files used at different stages of the workflow. All data processing, including statistical analysis, interpolation/extrapolation, and the enrichment of material records with elemental compositions, was implemented in R^72^ (version 4.5.1) using R Notebooks^71^. The notebooks generate self-documenting.pdf reports containing code, explanations, intermediate results, and figures, allowing users to follow and reproduce the full calculation chain. All R Notebooks and their corresponding.pdf outputs are openly available on GitHub and Zenodo at https://doi.org/10.5281/zenodo.17894289^70^.
